# Risk evaluation of fetal growth restriction by combined screening in early and mid-pregnancy

**DOI:** 10.12669/pjms.36.7.1988

**Published:** 2020

**Authors:** Bo Wang, Chunhua Zhang

**Affiliations:** 1 Bo Wang, Department of Gynecology, The Affiliated Huaian No.1 People’s Hospital of Nanjing Medical University, Huaian 223300, Jiangsu Province, China; 2 Chunhua Zhang, Department of Gynecology, Maternal and Child Healthcare Hospital, Huaian 223001, Jiangsu Province, China

**Keywords:** Early pregnancy, Mid-pregnancy, Fetal growth restriction

## Abstract

**Objectives:**

To assess risk of fetal growth restriction (FGR) by combined screening in early and mid-pregnancy.

**Methods:**

Pregnant women who received prenatal examinations and delivered in our hospital from January 2015 to January 2019 were selected and retrospectively analyzed. All women completed two ultrasonographic examinations during pregnancy, i.e. Down’s screening during early pregnancy (11-13 + 6 weeks) and prenatal color Doppler screening during mid-pregnancy (20-24 weeks). A total of 33 FGR cases were screened out, and there were 1,507 normal pregnant women. The clinical, ultrasonographic and serological indices in early and mid-pregnancy were recorded. When the false positive rate was 5%, logistic regression analysis and receiver operating characteristic (ROC) curve were used to evaluate the influencing factors and predictive values of individual and combined indices for FGR in corresponding gestational weeks. The sensitivity and specificity of the optimal cutoff value of each index as well as the combination of optimal predictive indices were found by the area under ROC curve (AUC).

**Results:**

When the false positive rate was 5% in the single-index screening during early pregnancy, the parity, BPD, AC, HC, and FL had statistical significances. Multivariate analysis showed that the parity and BPD had statistical significances. During mid-pregnancy, univariate analysis revealed that the parity, BMI, BPD, AC, HC, FL, UTA-PI, UTA-RI, UA-PI and UA-RI had statistical significances. BMI, AC, UTA-PI, UTA-RI, UA-PI and UA-RI had statistical significances in multivariate analysis. BMI, UTA-PI and UA-PI were risk factors for FGR, with UTA-PI being most dangerous. AUC for combined screening exceeded those for individual screenings. The best combined screening program was BPD in early pregnancy + BMI + AC + UTA-PI + UTA-RI + UA-PI + UA-RI in mid-pregnancy. The optimal cutoff value was 0.015, with the sensitivity of 83.1% and the specificity of 61.3%.

**Conclusion:**

The predictive efficiency of combined FGR screening in early and mid-pregnancy surpasses that of simple mid-pregnancy screening. It is recommended to use the integrated screening program in early and mid-pregnancy to predict FGR.

## INTRODUCTION

Fetal growth restriction (FGR) means that the estimated fetal weight is below the 10th percentile of normal standard for a corresponding gestational age. FGR is affected by maternal, fetal, placental and umbilical cord factors.^1^ For fetuses younger than 32 weeks, FGR is mainly related to maternal complications and placental factors.^2^ However, the placenta has not matured in early or mid-pregnancy. Besides observation of the placental morphology and location, other aspects cannot be easily monitored. The umbilical cord is also in the early stage of development, so its length and shape are hardly discernible even with ultrasonography. To this end, researchers have endeavored to increase the predictive rate of FGR by exploring maternal and fetal factors. Old age, preeclampsia and chronic diseases of pregnant women can all increase the risk of FGR.^3^

In the past 30 years, the diagnostic values of fetal abdominal circumference and fetal weight for FGR have been well acknowledged. Meanwhile, the American College of Obstetricians and Gynecologists believes that abdominal circumference reduction is one of the sensitive single indices for FGR. The fetus is small in early pregnancy, which causes errors in measurement. Therefore, biparietal diameter (BPD), abdominal circumference (AC), head circumference (HC) and femur length (FL) are mostly detected after mid-pregnancy, but the predictive effects of using one index alone remain unsatisfactory. Recently, ultrasonographic and serological indices have been combined. Through the study of 472 cases of 26- to 30-week pregnant women, Jia et al. found that when the false positive rate was 5%, the predictive sensitivities of umbilical artery-pulse index (UA-PI) and umbilical artery-resistance index (UA-RI) toward FGR were 40% and 44% respectively.^4^ PAPP-A and β-HCG, which have been clinically used in Down’s screening, may help the diagnosis and treatment of fetal malformations. At present, fundal height and fetal weight are commonly employed to assess the possibility of FGR worldwide, but a low body weight does not indicate pathological growth abnormalities.^5^ Besides, the measurement of fundal height and fetal weight is also affected by many factors, such as amniotic fluid volume, fetal respiratory movement, maternal obesity, individual fetal differences, and operator skills, inducing errors in prediction. Although the diagnostic value of fundal height is limited, it remains the only method of screening.

We herein aimed to conduct risk evaluation of FGR through combined screening of different indices in early and mid-pregnancy, so as to provide clinical evidence for early diagnosis.

## METHODS

Pregnant women who received prenatal examinations and delivered in our hospital from January 2015 to January 2019 were selected and retrospectively analyzed. All women completed two ultrasonographic examinations during pregnancy, i.e. Down’s screening during early pregnancy (11-13 + 6 weeks) and prenatal color Doppler screening during mid-pregnancy (20-24 weeks). This study has been approved by the ethics committee of our hospital on January 6^th^, 2015, and written informed consent has been obtained from all cases.

### Inclusion criteria

1) Women with singleton pregnancy who received prenatal examinations and delivered in our hospital; 2) women who received Down’s screening during early pregnancy (11-13 + 6 weeks) and prenatal color Doppler screening during mid-pregnancy (20-24 weeks); 3) women with complete clinical data, including MAP, fetal growth, ultrasonographic and serological indices in early pregnancy as well as fetal growth and ultrasonographic indices in mid-pregnancy. Exclusion criteria: 1) Women with multiple pregnancy; 2) women who did not participate in any of the two screenings in our hospital and those who did not receive prenatal examinations in our hospital; 3) women who lacked data concerning any of MAP, fetal growth, ultrasonographic or serological indices; 4) women with chromosomal abnormalities, administration of drugs such as aspirin during pregnancy, history of delivering infants younger than expected gestational ages, history of smoking and complications such as heart, liver, kidney and blood diseases together with gestational diabetes. Finally, 1,540 eligible pregnant women were enrolled.

### Methods

The database of the 1,540 collected pregnant women was established according to the data during hospitalization: medical card number, name, medical record number, diagnosis, gestational week, age, parity, heights and weights on the days of blood sampling for Down’s screening in early pregnancy and color Doppler screening in mid-pregnancy, chromosomal abnormalities, congenital fetal abnormalities, use of aspirin during pregnancy, as well as histories of delivering infants younger than expected gestational ages, diabetes, smoking and diseases of the heart, liver, kidney and hematological system. BMI values in early and mid-pregnancy were calculated. According to FGR diagnostic criteria^1^ and the criteria for normal pregnancy,^6^ 33 FGR cases were screened out, and there were 1,507 normal pregnant women.

### Measurement of MAP

In 11-13 + 6 weeks, the blood pressure of the right arm was measured three times by an electronic sphygmomanometer. MAP (mmHg) = [systolic pressure + (diastolic pressure × 2)]/3.

### Ultrasonographic indices

In the supine position, 20-24-week pregnant women were examined with Voluson E8 ultrasound machine (GE, USA) and 5 MHz convex array probe. The uterine artery was found about 2 cm horizontally to the lateral side of the cervix. The Doppler gain and scan speed were selected, and the sampling volume was 2 mm, with the sampling line in the direction of blood flow as much as possible (angle <30°), aiming to obtain a continuous and stable UTA spectrum. The PI and RI values of bilateral UTAs were measured. Afterwards, the placental attachment point was found to determine the umbilical artery spectrum within 5 cm, and UA-PI and UA-RI were detected. UTA-PI was measured using the same method in 11-13 + 6 weeks and in 20-24 weeks. The mean values of bilateral uterine arteries were recorded. As to fetal growth indices, a 3.5-5 MHz convex array probe was used, and the largest skull plane was selected according to the ultrasonographic diagnostic criteria to determine BPD.^7^ Fetal abdominal margin was selected to measure AC. HC was measured along the skull ring, and FL was detected from the femur head to shaft excluding epiphysis. The values in early and mid-pregnancy were measured.

### Serological indices

Venous blood (3 ml) was collected and coagulated at room temperature to separate the serum. Two serum samples were employed to detect the levels of β-HCG and PAPP-A with immunofluorescence assay (DELFIA reagent, Finland) using Cobas e411 analyzer (Roche, Switzerland).

### Numbering of predictive indices

Early pregnancy-related indices were marked with No. 1: i.e. UTA-PI1, BPD1, AC1, HC1, FL1, MAP1, β-HCG1 and PAPP-A1. Mid-pregnancy-related indices were marked with No. 2: i.e. UTA-PI2, UTA-RI2, UA-PI2, UA-RI2, BPD2, AC2, HC2 and FL2.

### Statistical Analysis

All data were analyzed by SPSS16.0 software. The categorical data were expressed as mean ± standard deviation (x ± s), and the categorical variables were represented as frequency. The categorical data were subjected to the t test, and the categorical variables were compared by the Chi-square test. logistic regression analysis and receiver operating characteristic (ROC) curve were used to assess the influencing factors (odds ratio (OR) reflected its risk factor and protective factor) and predictive values of individual and combined indices for FGR in corresponding gestational weeks. The sensitivity and specificity of the optimal cutoff of each index as well as the combination of optimal predictive indices were found by the area under ROC curve (AUC). P<0.05 was considered statistically significant. AUC≤0.7: low diagnostic accuracy; 0.7<AUC≤0.9: moderate accuracy; AUC>0.9: high accuracy. OR>1: Risk factor; OR<1: protective factor; OR = 1: ineffective index.

## RESULTS

When the false positive rate was 5%, FGR cases and normal pregnant women had significantly different BPD1, AC1, HC1, FL1, BMI2, BPD2, AC2, HC2, FL2, UTA-PI2, UTA-RI2, UA-PI2 and UA-RI2.The FGR group had higher parity, MAP1, β-HCG1, UTA-PI1, BMI2, UTA-PI2, UTA-RI2, UA-PI2 and UA-RI2 than those of the normal pregnancy group, whereas lower maternal age, BMI1, PAPP-A1 BPD1, AC1, HC1, FL1, BPD2, AC2, HC2 and FL2. When the false positive rate was 5%, the two groups had significantly different case numbers of premature delivery, natural labor, cesarean section and fetal survival (Table-I). One fetus in the FGR group died because the mother no longer felt fetal movement, which was disclosed by B-scan ultrasonography before admission. One fetus in the normal pregnancy group died of pulmonary infection upon birth through cesarean section for the mother suffering from high fever in the 31st pregnancy week.

**Table-I:** Baseline clinical data.

Clinical data	FGR group (n=33)	Normal pregnancy group (n=1507)	t/χ^2^	P
Fetal survival	32	1506	21.873	0.000
Premature delivery	9	38	66.862	0.000
Natural labor	13	961	8.287	0.004
Cesarean section	20	545		
Parity	0.31±0.10	0.20±0.12	5.226	0.000
BMI1 (kg/m^2^)	20.46±2.51	20.51±2.61	0.109	0.913
Maternal age (year)	28.21±3.82	28.24±3.79	0.045	0.964
MAP1 (mmHg)	82.36±8.13	81.69±7.96	0.478	0.633
BPD1 (mm)	19.64±2.51	20.64±2.59	2.217	0.027
AC1 (mm)	58.36±7.57	61.27±7.81	2.119	0.034
HC1 (mm)	68.59±8.41	71.91±8.40	2.246	0.025
FL1 (mm)	7.25±1.82	7.92±1.89	2.016	0.044
UTA-PI1	1.95±0.32	1.94±0.34	0.167	0.867
PAPP-A1 (IU/L)	4.48±0.17	4.51±0.15	1.133	0.257
β-HCG1 (IU/L)	72.10±6.43	71.91±5.91	0.182	0.855
BMI2 (kg/m^2^)	21.56±2.91	20.34±2.63	2.630	0.009
BPD2 (mm)	52.44±3.51	53.79±3.67	2.092	0.037
AC2 (mm)	160.02±5.71	167.25±6.03	6.821	0.000
HC2 (mm)	186.79±5.25	192.08±5.71	5.273	0.000
FL2 (mm)	35.11±2.76	36.14±2.80	2.091	0.037
UTA-PI2	1.12±0.23	0.91±0.23	5.189	0.000
UTA-RI2	0.59±0.11	0.55±0.08	2.815	0.005
UA-PI2	1.30±0.24	1.22±0.18	2.505	0.012
UA-RI2	0.73±0.06	0.70±0.06	2.841	0.005

Multivariate logistic regression analysis of single indices during early pregnancy revealed that when the false positive rate was 5%, parity and BPD1 had statistical significance for FGR prediction, with ORs of 1.565 (1.025-2.680) and 0.854 (0.772-0.945), respectively. Besides, parity was a risk factor for FGR (Table-II).

**Table-II:** Multivariate logistic analysis results of single indices during early pregnancy for FGR prediction

Screening index	P	OR	Range of OR
Parity	0.039	1.656	1.025-2.680
BPD1	0.001	0.853	0.772-0.945

Multivariate logistic analysis of single indices during mid-pregnancy revealed that when the false positive rate was 5%, BMI2, AC2, UTA-PI2, UTA-RI2, UA-PI2 and UA-RI2 were included in the model, of which BMI2, AC2 and UA-PI2 were risk factors for FGR and UTA-PI2 had the highest risk (Table-III).

**Table-III:** Multivariate logistic analysis of single indices during mid-pregnancy for FGR prediction.

Screening index	P	OR	Range of OR
BMI2	0.001	1.134	1.047-1.231
AC2	0.000	0.950	0.932-0.976
UTA-PI2	0.000	340.19	29.276-3946.545
UTA-RI2	0.001	0.000	0.000-0.004
UA-PI2	0.000	87.198	8.266-950.886
UA-RI2	0.000	0.000	0.000-0.039

Based on the above results, parity and BMI2 were used as clinical factors, while BPD1, AC2, UTA-PI2, UTA-RI2, UA-PI2 and UA-RI2 were utilized as ultrasonographic factors. Afterwards, the eight factors were randomly combined freely and subjected to logistic regression analysis. Then ROC curves were plotted to obtain AUCs. As listed in Table-IV, the best screening program for early and mid-pregnancy is BPD1 + BMI2+ AC2 + UTA-PI2 + UTA-RI2 + UA-PI2 + UA-RI2 with an AUC of 0.789 (95%CI: 0.732-0.847), exceeding that of the best screening program for early pregnancy (parity + BPD1; AUC: 0.622; 95%CI: 0.549-0.694) and that of best screening program for mid-pregnancy (BMI2 + AC2 + UTA-PI2 + UTA-RI2 + UA-PI1 + UA-RI2; AUC: 0.775; 95%CI: 0.716-0.835). The optimal cutoff of combined screening program in early and mid-pregnancy is 0.015, with the sensitivity of 83.1% and the specificity of 61.3% as shown in Table-V.

**Table-IV:** AUCs of best screening programs for FGR in early, mid- as well as early and mid-pregnancies.

Best screening program	AUC	95%CI
Early pregnancy (parity + BPD1)	0.622	0.549-0.694
Mid-pregnancy (BMI2 + AC2 + UTA-PI2 + UTA-RI2 + UA-PI2 + UA-RI2)	0.775	0.716-0.835
Combined screening in early and mid-pregnancy (BPD1 + BMI2 + AC2 + UTA-PI2 + UTA-RI2 + UA-PI2 + UA-RI2)	0.789	0.732-0.847

**Table-V:** Optimal cutoff values, sensitivities and specificities of best screening programs for FGR in early, mid- as well as early and mid-pregnancies.

Best screening program	Cutoff	Sensitivity	Specificity
Early pregnancy (parity + BPD1)	0.027	44.6%	78.2%
Mid-pregnancy (BMI2 + AC2 + UTA-PI2 + UTA-RI2 + UA-PI2 + UA-RI2)	0.019	73.8%	69.8%
Combined screening in early and mid-pregnancy (BPD1 + BMI2 + AC2 + UTA-PI2 + UTA-RI2 + UA-PI2 + UA-RI2)	0.015	83.1%	61.3%

## DISCUSSION

FGR is currently diagnosed using the ACOG guidelines,^1^ but the reason for the lower 10th percentile of normal weight of the same gestational age remains unclear. At present, FGR is mainly symptomatically treated. For example, low molecular weight heparin and magnesium sulfate can improve the fetal prognosis.^8^

FGR is mostly found by monitoring the body weight through B-scan ultrasonography, using fetal BPD, HC, AC and FL as crucial indices. Butt et al. reported that AC was the best single index for predicting FGR.^9^ Consistently, multivariate regression analysis in this study showed that BPD had the best predictive effect in early pregnancy, and AC was the best single index in mid-pregnancy. We herein also found that MAP in early pregnancy had no statistical significance for FGR prediction.

In addition, PAPPA-A and β-HCG in early pregnancy were not significantly different between FGR and normal pregnancy groups. Yu et al. reported that AUCs of PAPP-AMOM and β-HCGMOM for FGR prediction were 0.83 (0.67-1.00) and 0.94 (0.80-1.16), respectively.^10^ Keikkala et al. found that the predictive rate of β-HCG for FGR in mid-pregnancy was lower than 50%.^11^ In this study, β-HCG or PAPP-A had no predictive value for FGR, which may be attributed to the low number of involved FGR cases.

The umbilical artery was a key channel for maternal-fetal nutrient exchange. In normal pregnancy, with increasing gestational age, UA, UTA PI and RI reduce,^6^ However, we found that the blood flow parameters of umbilical artery and uterine artery increased upon FGR. This result is consistent with those of Jia et al.^4^ and O’Dwyer et al.,^12^ indicating that FGR may be related to UA and UTA. In this study, FGR was not significantly related with UTA-PI in early pregnancy, but related with UA and UTA in mid-pregnancy. It is well-established that parity, maternal race, age, socioeconomic level, living environment, smoking, drinking and pregnancy complications are risk factors for FGR.^13^ This study showed that parity and BMI in mid-pregnancy were associated with FGR.

Predicting FGR by using a combination of indices has seldom been referred hitherto. Korkalainen et al. studied 72 pregnant women with FGR, and found that a combination of umbilical artery blood flow parameters had predictive value for FGR. Pulsation index had the highest predictive efficiency.^14^ Similarly, Odibo et al. reported that combining umbilical arterial blood flow indices with others such as intravenous catheterization predicted FGR better than single indices did.^15^ This study combined clinical factors, fetal growth indices, MAP, ultrasonographic indices and serological indices. The combined screening program in early and mid-pregnancy had a higher AUC (0.789) than those of early pregnancy (0.622) and mid-pregnancy (0.775). Nevertheless, the predictive value of combined screening in early and mid-pregnancy was not significantly higher than that in mid-pregnancy, probably because of the statistical correlations of BPD in early pregnancy with BMI, AC, UA-PI and UA-RI in mid-pregnancy. Furthermore, the optimal cutoff of early and mid-pregnancy was 0.015, the sensitivity was 83.1% and the specificity was 61.3%. Hence, the overall efficacy exceeded those of early pregnancy and mid-pregnancy, suggesting higher clinical applicability.

### Limitations of the study

It is a retrospective single-center study, and the sample size is small. In the future, we will perform more prospective multicenter studies with large sample sizes.

## CONCLUSION

In summary, the predictive efficiency of combined FGR screening in early and mid-pregnancy surpasses that of simple mid-pregnancy screening. It is recommended to use the integrated screening program in early and mid-pregnancy to predict FGR.

### Authors’ Contributions:

**CZ:** Study design and significant manuscript revision.

**BW:** Manuscript drafting, clinical data collection and analysis.

**BW & CZ:** Approval of manuscript submission.

All authors are responsible and accountable for the accuracy or integrity of the work.
